# Pinocembrin: A Novel Natural Compound with Versatile Pharmacological and Biological Activities

**DOI:** 10.1155/2013/379850

**Published:** 2013-08-05

**Authors:** Azhar Rasul, Faya Martin Millimouno, Wafa Ali Eltayb, Muhammad Ali, Jiang Li, Xiaomeng Li

**Affiliations:** ^1^The Key Laboratory of Molecular Epigenetics of MOE, Institute of Genetics and Cytology, Northeast Normal University, Changchun 130024, China; ^2^Dental Hospital, Jilin University, Changchun 130041, China; ^3^Institute of Molecular Biology and Biotechnology, Bahauddin Zakariya University, Multan 60800, Pakistan

## Abstract

Pinocembrin (5,7-dihydroxyflavanone) is one of the primary flavonoids isolated from the variety of plants, mainly from *Pinus* heartwood, *Eucalyptus, Populus, Euphorbia*, and *Sparattosperma leucanthum*, in the diverse flora and purified by various chromatographic techniques. Pinocembrin is a major flavonoid molecule incorporated as multifunctional in the pharmaceutical industry. Its vast range of pharmacological activities has been well researched including antimicrobial, anti-inflammatory, antioxidant, and anticancer activities. In addition, pinocembrin can be used as neuroprotective against cerebral ischemic injury with a wide therapeutic time window, which may be attributed to its antiexcitotoxic effects. Pinocembrin exhibits pharmacological effects on almost all systems, and our aim is to review the pharmacological and therapeutic applications of pinocembrin with specific emphasis on mechanisms of actions. The design of new drugs based on the pharmacological effects of pinocembrin could be beneficial. This review suggests that pinocembrin is a potentially promising pharmacological candidate, but additional studies and clinical trials are required to determine its specific intracellular sites of action and derivative targets in order to fully understand the mechanism of its anti-inflammatory, anticancer, and apoptotic effects to further validate its medical applications.

## 1. Introduction

Throughout the history of civilization, natural products have served human beings as a primary source of medicine [1]. The term “natural products” comprises chemical compounds that are derived from living organisms such as plants, fungi, bread molds, microorganisms, marine organisms, and terrestrial vertebrates and invertebrates [2]. In 2008, of the 225 drugs being developed, 164 were of natural origin, with 108 being derived from plants, 25 from bacterial sources, 7 from fungal, and 24 from animal sources, and, to throw some more numbers around, of the 108 plant-based drugs, 46 were in preclinical development, 14 in phase I, 41 in phase II, 5 in phase III, and two had already reached preregistration stage [3]. An analysis of medical indications by resource of compounds has established that natural products and related drugs, including anticancer, antibacterial, antiparasitic, anticoagulant, and immunosuppressant agents, are being used to treat 87% of all categorized human diseases [4]. Plants provide an extensive reservoir of natural products, demonstrating important structural diversity, and offer a wide variety of novel and exciting chemical entities in modern medicine [2, 4–9]. Historical experiences with plants as therapeutic tools have led to discoveries of many important, effective, and novel drugs including older drugs such as quinine and morphine and newer drugs such as paclitaxel (taxol), camptothecin, topotecan, and artemisinin [10]. 

The significance of natural products in health care is supported by a report that 80% of the global population still relies on plant derived medicines to address their health care needs [11]. It is also reported that 50% of all drugs in clinical use are natural products, or their derivatives, or their analogs [12], and 74% of the most important drugs consist of plant-derived active ingredients [13]. Until the 1970s, drug discovery was based on screening of a large number of natural and synthetic compounds, with the advent of computer and other molecular biology techniques, resulting in the modern and rational drug discovery [14]. Plant-based drugs have provided the basis of traditional medicine systems that have been employed in various countries such as Egypt, India, and China since prehistoric times [12]. The medicinal properties of plants have been documented already on Assyrian clay tablets dated about 2000 B.C. and reported in the Egyptian culture, the Indian Ayurveda [15], and traditional Chinese medicines (TCMs) [16].

All this said is implying that natural products including plants are important and valuable resources for drug development of natural origin [17]. Furthermore, a large number of natural compounds have been reported, which have been isolated from plants possessing wide variety of biological functions such as total glucosides of astragalus showing anti-inflammatory activity, tripterygium wilfordii multiglycoside, sinomenine [18], and camptothecin, taxol, vinblastine, vincristine, podophyllotoxin, and colchicine that demonstrate antineoplastic activity [19]. Indeed, molecules derived from natural sources including plants, marine organisms, and microorganisms have played and continue to play a dominant role in the discovery of leads for the development of conventional drugs for the treatment of the majority of human diseases. Chemoprevention was defined as the administration of agents to prevent induction, to inhibit, or to delay the progression of disease [20]. Mainly several scientific studies have been carried out on *Euphorbia hirta* Linn., widely spread in south China, which is extensively used in folk Chinese medicines for several ailments such as dysentery, eczema, hematuria, hypersensitivity, and gastroenteritis [21]. In addition, many studies have also reported that natural products have antimicrobial [22, 23], anticancer [24, 25], antioxidant [26, 27], anti-inflammatory [28, 29], and antifungal properties [30, 31]. The yield extract of leaves of *Sparattosperma leucanthum *(Vell.) K. Schum, that is, a native tree of Brazil, is popularly known as “caroba branca” or “ipê branco.” Previous phytochemical studies on the genus *Sparattosperma* described the isolation of the flavanone pinocembrin-7-O-(-d-neohesperidoside). Pinocembrin, one of the most important phytochemicals among flavonoids, acts as anti-inflammatory, antimicrobial, and antioxidant agent [24, 26, 32]. The extensive research indicated that pinocembrin has potential biological activities, which have made further interest among the chemists and biologists.

This review summarizes the recent researches on pinocembrin focusing on its biological and pharmacological activities. The literature was screened through various e-sites including PubMed, Scopus, and Elsevier Science Direct. Access to the Elsevier Science Direct Journals was made possible through the library of Northeast Normal University, Changchun, China. The searched literature mainly focused on recent advances, and additional manual searches were carried out on relevant medical journals and the google search Engine. Key words used for search were “pinocembrin,” “pinocembrin and biological activity,” “anticancer activity,” “inflammatory activity,” “cytotoxicity,” and “medicinal plants.” The data collected from primary sources and/or from data that superseded earlier work were included. 

## 2. Natural Sources of Pinocembrin

**Figure 1:**
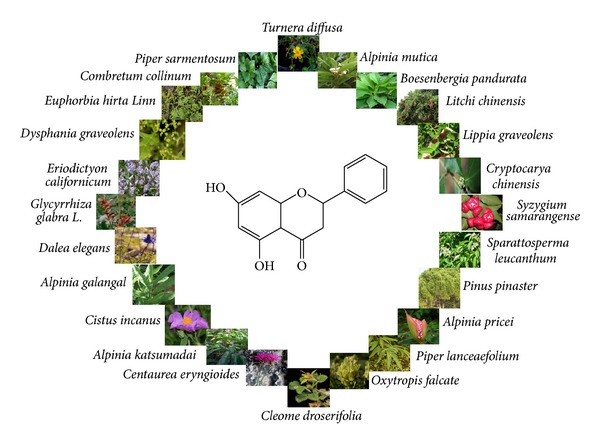
Chemical structure and natural sources of pinocembrin.

Pinocembrin (Figure 1) has been identified in several plants such as the numerous genera of the *Piperaceae* family, which comprises fourteen genera and 1950 species that are reported as the rich source of pinocembrin. Of which, two genera, *Peperomia* and *Piper*, have been proved to be the most widespread and most diverse with 600 and 700 species, respectively [30, 33]. In addition to this family, pinocembrin has been also isolated from plants of Lauraceae and Asteraceae families, which comprise a large number of species. Of which, about 250 species of genus *Cryptocarya* are mainly distributed in tropical and subtropical regions, and 600 species of *Helichrysum *are located in Africa, of which some 244 species are found in South Africa [32]. Pinocembrin was also isolated from aerial parts of *Flourensia oolepis* S.F. Blake (Asteraceae) [34] and honey [35]. Further, pinocembrin, being a flavonoid natural compound, is located in fruits, vegetables, nuts, seeds, herbs, spices, stems, flowers, tea, and red wine [36, 37]. It has also shown a variety of pharmacological properties of interest in the therapy of several diseases including inflammation by inhibiting bacterial colonization, cancer, or vascular ailments [38, 39]. The summary of plants containing pinocembrin, parts used, and biological/pharmacological activities is shown in Table 1. As shown in Figure 1, accumulated data indicate that pinocembrin was isolated from many plant species such as *Alpinia mutica *[40, 41], *Litchi chinensis* [42], *Lippia graveolens *[43], *Lippia origanoides *[44, 45], *Dalea elegans *[46], *Oxytropis falcate *[47, 48], *Glycyrrhiza glabra L.* [49, 50], *Sparattosperma leucanthum *[51], *Cleome droserifolia *[52], *Lychnophora markgravii *[53], *Helichrysum gymnocomum *[54],* Syzygium samarangense *[55], *Centaurea eryngioides *[56], *Cistus incanus* [27],* Turnera diffusa* [57], and *Eriodictyon californicum *[58]. 

Apart from natural sources, it has been noted that pinocembrin can be biosynthesized. The strategy to produce pinocembrin, a flavanone, by microorganisms was to design and express an artificial phenylpropanoid pathway. This was accomplished by assembling of phenylalanine ammonia-lyase (PAL) from the yeast *Rhodotorula rubra*; 4-coumarate: CoA ligase (4CL) from the actinomycete *S. coelicolor*; chalcone synthase (CHS) from the licorice plant *Glycyrrhiza echinata*; and chalcone isomerase (CHI) from the plant *Pueraria lobata* on a single pET plasmid in *E. coli* [37–39, 59–61].

## 3. Biological Activity of Pinocembrin and Mechanisms of Action

The biological activity of natural compounds is generally investigated with emphasis on the mechanisms of actions. Several studies have been conducted *in vitro* and *in vivo* to determine the biological properties ascribed to pinocembrin and to elucidate its mechanisms of actions. In this case, some researchers pointed out the effect of functional groups on the biological activity of certain molecules to evaluate the effect of hydroxyl group on biological activity of pinocembrin and its analogues.

### 3.1. Antibacterial Activity

For centuries, natural products, including pinocembrin, have been used to treat microbial infections. Drewes and van Vuuren [54] investigated the antibacterial effect of pinocembrin with three kinds of Gram-negative bacteria (*E. coli*, *P. aeruginosa*, *and K. pneumoniae*) and three kinds of Gram-positive bacteria (*B. subtilis*, *S. aureus*, and* S. lentus*) by measuring the minimal inhibitory concentrations in microgram of DMSO extract (mg of extract/mL) determined by an adjustment of the agar streak dilution method based on radial diffusion. Another investigation was conducted to evaluate the effect of pinocembrin by the metabolic engineering technique for the production in bacteria under cultural conditions which were *E. coli* at a cell density of 50 g/L, incubated in the presence of 3 *μ*M tyrosine or phenylalanine; the yields of pinocembrin reached about 60 mg/L. Phenylalanine ammonia lyase (PAL) from the yeast *Rhodotorula rubra*, 4-coumarate: CoA ligase (4CL) from an actinomycete *Streptomyces coelicolor*, and chalcone synthase (CHS) from a licorice plant *Glycyrrhiza echinata*, taken individually are each an active ingredient for fermentation production of flavanones; such as pinocembrin in *Escherichia coli* via different pathway including phenylpropanoid pathway. In the construction of set, they are placed in order under the control of pT7 promoter and the ribosome binding sequence (RBS) in the pET vector. These pathways bypassed cinnamate-4-hydroxylase (C4H), a cytochrome P-450 hydroxylase, because the bacterial 4CL enzyme legated coenzyme A to both cinnamic acid and 4-coumaric acid. *E. coli* cells containing the gene clusters produced two flavanones. The fermentative production of flavanones in *E. coli* is the sine qua non provided in the construction of a library of unnatural flavonoids in bacteria [37, 60, 61].

The mechanisms of actions of pinocembrin were studied to evaluate its effect on the bacterial membranes of *Neisseria gonorrhoeae, E. coli, P. aeruginosa, B. subtilis, S. aureus, S. lentus, *and* K. pneumoniae* by observing changes in membrane composition and monitoring the metabolic engineering technique, which revealed that pinocembrin induced cell lysis through a metabolic engineering technique [37, 60–62].

### 3.2. Anti-Inflammatory Activity

Although the type of inflammatory responses may differ among diseases, inflammation and disease conditions are linked through the production of inflammatory mediators by macrophages and neutrophils. Overexpression activity of the enzyme cyclooxygenases- (COX-) 1 and COX-2 produces inflammatory mediators such as prostaglandin E 2 (PGE 2). Anti-inflammatory drugs together with nonsteroidal anti-inflammatory drugs (NSAIDs) suppress the inflammatory response by inhibiting infiltration and activation of inflammatory cells as well as their synthesis or, secondly, release of mediators or effects of inflammatory mediators themselves [63].

The anti-inflammatory activity of pinocembrin against sheep red blood cell-induced mouse paw oedema as a model of delayed-type hypersensitivity reaction *in vitro* and in the mouse model of LPS-induced acute lung injury inhibited significantly enzymatic and nonenzymatic lipid peroxidation (IC_50_ = 12.6 and 28 *μ*M, resp.) [28]. Pulmonary edema, histological severities, and neutrophil, lymphocyte, and macrophage infiltration increased by LPS administration; this would mean that pinocembrin exhibited anti-inflammatory activity in the sheep red blood cell-induced delayed-type hypersensitivity reaction. Although it downregulated TNF-*α*, IL-1*β*, and IL-6 and significantly suppressed I*κ*B*α*, JNK, and p38MAPK with (20 or 50 mg/kg, i.p.) in LPS-induced lung injury, having regard to the foregoing, pinocembrin is a natural compound recommended for the modulation of inflammatory responses [28, 29, 64].

### 3.3. Antimicrobial Activity

Flavonoid compounds in general and in particular pinocembrin are well-known plant compounds that have antimicrobial and anti-inflammatory properties [65]. Scientists and clinicians have demonstrated *in vitro* and *in vivo* the biological or pharmacological properties of pinocembrin and have elucidated mechanisms of action [23]. In this momentum, production of glucosyltransferase from microorganisms according to the results obtained on *Staphylococcus aureus; Escherichia coli, Candida albicans, Bacillus subtilis, Candida albicans, Trichophyton mentagrophytes, Streptococcus mutans, Neisseria gonorrhoeae*, treatment with pinocembrin at daily doses of 100 mg/kg body weight the animals as well as the controls died between the 6th and 24th day after beginning. The possible mechanisms of the antimicrobial action of pinocembrin demonstrated the highest inhibition of the enzyme activity, and growth of the bacteria indicates that pinocembrin inhibited 100% of the *Neisseria gonorrhoeae* panel at 64 g/mL and 128 g/mL, respectively, whereas cyclolanceaefolic acid methyl ester inhibited 44% of the strains at 128 g/mL [22, 66–68].

### 3.4. Anticancer Activity

Due to the toxic effects of synthetic drugs, accumulated data indicate that prevailing treatment options have limited therapeutic success in human cancers; therefore, there is considerable emphasis on identifying novel natural products that selectively induce apoptosis and growth arrest in cancer cells without cytotoxic effects in normal cells [69]. Apoptosis is defined as an extremely synchronized mode of cell death and is characterized by distinct morphological features, including cell membrane blabbing, chromatin condensation, and nuclear fragmentation [70, 71]. The normal cell regulation and during disease conditions the importance of signaling has been recognized, [72, 73] and many well-known targets at the signaling levels have been identified that are critical rapid proliferate of cancer cells. It is believed that in normal cells, certain cellular signals control and regulate their growth and all growth mechanisms, and when these signals are altered due to various mutations that prevent cells to undergo apoptosis, normal cells are transformed into cancerous cells and undergo hyperproliferation. Therefore, to arrest cancerous cell proliferation, regulation of apoptosis plays a critical role [74–76]. Accumulated data suggest that various anticancer chemopreventive agents can induce apoptosis which causes death in cancerous cells [77–84]. Although several studies revealed that pinocembrin can inhibit, delay, block, or reverse the initiation; promotional events associated with carcinogenesis are needed for the prevention and/or treatment of cancer. Here, we reviewed studies related to anticancer activity of pinocembrin to allow scientists and researchers to have a clearer view of this natural compound.

Based on the research anterior made, pinocembrin has shown cytotoxicity against certain cancer cell lines such as colon cancer cell line (HCT116), with relatively less toxicity toward human umbilical cord endothelial cells [24]. In colon cancer cell line (HCT116), pinocembrin increased increased the activity of heme oxygenase, caspase-3 and -9, and mitochondrial membrane potential (MMP) but did not affect the activities of cytochrome P450 reductase, quinone reductase, UDP glucuronosyltransferase, and glutathione-S-transferase [24, 25]. Although some *in vivo* and *in vitro* studies reveal that pinocembrin can promote the differentiation of EPCs and improve the biological functions in rat liver micronucleus and medium-term carcinogenicity; interestingly, pinocembrin slightly increased the number of GST-P positive foci, PI3 K-eNOS-NO signaling pathway when given prior to diethyl-nitrosamine injection, and adhesion of EPCs. The effect of pinocembrin may help to protect against chemical-induced hepatocarcinogenesis and suggest that the promoting effect of this compound might be due to lipid peroxidation [85]. The details of all the information regarding the molecular targets of pinocembrin in different cancer types are recorded in Table 2. 

### 3.5. Antifungal Activity

Microbial infections especially fungal are a common public health problem ranging from superficial to deep infections. The superficial mycoses sometimes reach high endemic levels, especially in tropical areas. The treatment of fungal infections or mycoses is becoming more and more problematic due to the development of antimicrobial resistance to some kind of drugs. It is for that reason the natural products have been used to treat these infections and to demonstrate the ability to inhibit the growth of various pathogens agents. The antimicrobial activity against *P. italicum* and *Candida albicans*, with a minimal inhibitory concentration value of 100 microg/mL, shows that pinocembrin exhibited antifungal activity and inhibited the mycelial growth of *P. italicum* by interfering energy homeostasis and cell membrane damage of the pathogen [30, 31]. 

### 3.6. Neuroprotective Activity

The diverse array of bioactive nutrients present in the natural products plays a pivotal role in prevention and cure of various neurodegenerative diseases, such as Alzheimer's disease (AD), Parkinson's disease, and other neuronal dysfunctions [86]. Cerebral ischemic injury is a debilitating disease that can occur with great morbidity, during asphyxiation, shock, brain injury, extracorporeal circulation, and cardiac arrest [87, 88]. The neuroprotective effects of naturally occurring compound, pinocembrin, are being evaluated in this review. Previous studies demonstrated that pinocembrin can be used as neuroprotective against cerebral ischemic injury with a wide therapeutic time window, which may be attributed to its antiexcitotoxic effects [89] and decreased glutamate-induced SH-SY5Y cell injury and primary cultured cortical neuron damage in oxygen-glucose deprivation/reoxygenation (OGD/R). Pinocembrin alleviates cerebral ischemic injury in the middle cerebral artery occlusion rats [90–92] and also enhanced cognition by protecting cerebral mitochondria structure and function against chronic cerebral hypoperfusion in rats [93]. In another attempt to understand the mechanism of action of pinocembrin, it increased ADP/O, glutathione, state 3 respiration state, neuronal survival rates, and oxidative phosphorylation rate in NADH/FADH2 and decreased LDH release, reactive oxygen species, nitric oxide, neuronal nitric oxide synthase (nNOS), inducible NOS (iNOS), and 4 respiration state (V4) in NADH. Moreover, pinocembrin enhanced ATP content in brain mitochondria in SH-SY5Y cells; DNA laddering and caspase-3 are downregulated and increased PARP degradation [89, 94] and resulted in the alleviation of brain injury in the global cerebral ischemia/reperfusion (GCI/R) rats [94]. Furthermore, pinocembrin decreased neurological score and reduced brain edema induced by GCI/R. Pinocembrin also lessened the concentrations of Evan's blue (EB) and fluorescein sodium (NaF) in brain tissue of the GCI/R rats and alleviated the ultrastructural changes of cerebral microvessels, astrocyte end-feet, and neurons and improved cerebral blood flow (CBF) in the GCI/R rats. In addition, pinocembrin increased the viability and mitochondrial membrane potential of cultured rat cerebral microvascular endothelial cells (RCMECs) induced by oxygen-glucose deprivation/reoxygenation (OGD/R) [95]. Therefore, pinocembrin may be a novel therapeutic strategy to reduce cerebral ischemia [89, 94].

## 4. Conclusions and Future Perspectives

This review suggests that pinocembrin is a good pharmacological drug with potential antioxidative, anti-inflammatory, antitumor, and antimicrobial properties. Several research results demonstrated the potential applications of pinocembrin both* in vitro* and *in vivo*. Pinocembrin is a natural product with a small molecular weight and is a biologically active constituent of honey, an edible nutrient, which ensures safety of pinocembrin during long-term administration, combined with its cost and future therapeutic potential, making it an ideal therapeutic agent. Pinocembrin analogues with improved pharmacokinetic and pharmacodynamics may also encourage further advances. Many studies have shown that pinocembrin induces apoptosis of many types of cancer cells, but mechanisms of actions have not been fully elucidated. This review suggests that pinocembrin may establish direct medicinal application as a pharmaceutical agent or may serve as chemical templates for the design, synthesis, and semisynthesis of new substances for the treatment of human diseases. Additional studies and clinical trials are required to determine its specific intracellular sites of action and derivative targets in order to fully understand the mechanisms of its anti-inflammatory, anticancer, and apoptotic effects to further validate this compound in medical applications and to make clear the potential role of pinocembrin as a medicinal agent in the prevention and treatment of various diseases.

## Figures and Tables

**Table 1:** Plants containing pinocembrin with their mode of actions.

Plants name		Part used/extract	Functions	References
Botanical name	Common name			
*Alpinia mutica *	Orchid ginger	Air-dried Rhizome	Antiplatelet, antioxidant	[40, 41]
*Alpinia katsumadai *	Katsumadai	Seeds	Antibacterial, antiinflammatory	[96–99]
*Alpinia pricei *	Prospero Alpini	Roots	Antiinflammatory	[100, 101]
*Alpinia galangal *	Siamese ginger	Roots	Anticancer	[24]
*Alpinia rafflesiana *	Raffles' alpinia	Ripe fruits	DPPH free radical scavenger	[102]
*Boesenbergia pandurata *	Ginger	Fingerroot Rhizome	Antiinflammatory, antioxidant	[25, 85, 103, 104]
*Centaurea eryngioides *	Centory	—	Antitumor	[56]
*Cleome droserifolia *	Black thorn/egy	Aerial parts	Antirheumatic Antifever and antiinflammation	[52]
*Combretum collinum *	Combretum	Pulverized leaves	Antimicrobial, antimalarial	[105]
*Cryptocarya chinensis *	—	Air-dried Leaves	Antituberculosis	[106]
*Cryptocarya konishii *	Brown Laurel	Woods	Antibacterial, anticancer	[107]
*Cystus incanus *	—	—	Antioxidant/antiestrogenic	[27]
*Dalea elegans *	Prairie clover/indigo bush	Roots	Antibacterial	[46]
*Dysphania graveolens *	Fetid goosefoot	—	Antimicrobial, larvicidal, hepatoprotective, antihyperlipidaemic	[108]
*Eriodictyon californicum *	Yerba santa	Leaves	Chemopreventive agents	[58]
*Euphorbia hirta Linn *	Asthma herb	Aerial part	Antitumour, antifilarial	[109]
*Glycyrrhiza glabra L. *	Liquorice	Aerial parts	Cognitive functions, cholinesterase activity	[49, 50]
*Helichrysum gymnocomum *	—	Flowers	Antimicrobial	[54]
*Lippia graveolens *	Oregano		Antigiardial	[43]
*Lippia origanoides *	Wild marjoram	Flowers, leaves, stems	Antimicrobial	[44, 45]
*Litchi chinensis *	Lychee	Seeds		[42]
*Lychnophora markgravii *	—	Aerial parts	Antileishmania	[53]
*Oxytropis falcate *	—	Whole plants	Antipain, antiarthritis	[47, 48]
*Piper chimonantifolium *	—	Leaves	Antifungal	[62, 110]
*Piper lanceaefolium *	—	Leaves	Antibacterial	[30, 62]
*Piper sarmentosum *	—	Aerial parts	Antifeedant, anticarcinogenic	[111]
*Sparattosperma leucanthum *	—	Leaves	—	[51]
*Syzygium samarangense *	Champoo	Pulp, seeds of the fruits	Antioxidants	[55]
*Turnera diffusa *	Damiana	Leaves	Antiaromatase	[57]

**Table 2:** Molecular targets of pinocembrin in different cancer types.

Cancer types	Cell lines	IC_50_/concentration	Major targets	References
Colon	HCT-116, HT-29	26.33 to 143.09 *µ*g mL^−1^ 1.6 to 13.6 *μ*M	Superoxide anion radical↓, Bax↑, NO_2_↓, ΔΨm↓	[24, 111, 112]
Leukaemia	HL-60	IC_50_ < 100 ng/mL	Fas↑, FasL↑, caspase-3/8/9↑, tBid↑	[100, 113]

↓: downregulation; ↑: upregulation.
